# 16886 Moyamoya-Like Vasculopathies Observed In a Novel Mouse Surgical Model

**DOI:** 10.1017/cts.2021.458

**Published:** 2021-03-30

**Authors:** Justin F. Fraser, Laura Whitnel, Jill Roberts

**Affiliations:** University of Kentucky

## Abstract

ABSTRACT IMPACT: Development of our animal model of moyamoya will provide a meaningful assessment of therapeutic efficacy of interventions applicable to the clinical setting. OBJECTIVES/GOALS: Moyamoya is a cerebrovascular condition with progressive stenosis of the internal carotid arteries (ICA) and formation of abnormal vascular collaterals at the base of the brain, all of which result in ischemic and hemorrhagic strokes. We aim to develop a needed animal model of this condition in order to develop new therapeutics. METHODS/STUDY POPULATION: Male and female C57Bl/6J mice (4 months old) underwent surgery for the unilateral placement of a microcoil (0.16 mm ID) onto the proximal ICA or sham control. After 30 and 60 days (N = 6-8/time point), the brain blood vessels were examined for changes in diameter, number of anastomoses, and development of new collaterals using DiI stain. Brain tissue was examined for micro-hemorrhages using Prussian blue stain, and cross-sections of blood vessels were examined for intimal thickening using H&E and smooth muscle actin. Expression of vascular endothelial growth factor (VEGF), which is associated with angiogenesis and moyamoya syndrome, was quantified by qPCR. Blood samples were also analyzed for inflammatory biomarkers using ELISA. RESULTS/ANTICIPATED RESULTS: Within 30 days, the distal ICA and anterior cerebral artery (ACA) had significantly decreased diameters at the Circle of Willis, with an initial decrease in the number of cortical anastomoses. Histology demonstrated smaller lumen diameter and alterations to in the various layers of the blood vessels, indicating intimal thickening and stenosis of the affected blood vessels. There was also a significant increase in the number of intracranial micro-bleeds, suggesting a compromised vascular integrity. This may be due, in part, to a significant upregulation in VEGF gene expression within the striatum, a region of hemorrhagic occurrence in moyamoya patients. DISCUSSION/SIGNIFICANCE OF FINDINGS: We report the development of an animal model with vasculopathies that mimic those observed in patients with moyamoya syndrome. With further characterization, this animal model will have a positive impact as a meaningful assessment of therapeutic efficacy of interventions applicable to the clinical setting.

